# The trajectory of patient‐reported outcomes after hip preservation surgery: A National Registry Study

**DOI:** 10.1002/ksa.12771

**Published:** 2025-08-19

**Authors:** Junya Yoshitani, Seper Ekhtiari, Ajay Malviya, Vikas Khanduja

**Affiliations:** ^1^ Young Adult Hip Service, Department of Trauma and Orthopaedics Addenbrooke's ‐ Cambridge University Hospitals NHS Foundation Trust Cambridge UK; ^2^ Division of Orthopaedic Surgery, Sinai Health University of Toronto Toronto Ontario Canada; ^3^ Department of Trauma and Orthopaedic Surgery, Northumbria Hip Preservation Unit Northumbria Healthcare NHS Foundation Trust Cambridge UK; ^4^ Division of Trauma and Orthopaedics, Department of Surgery University of Cambridge Cambridge UK

**Keywords:** hip preservation surgery, hip arthroscopy, periacetabular osteotomy, patient‐reported outcome measures, post‐operative trajectory

## Abstract

**Purpose:**

Understanding the trajectory of postoperative patient‐reported outcomes after hip preservation surgery is essential. This study aims to analyse patient‐reported outcome trajectories up to 2 years post‐surgery using the UK's national hip preservation registry and to examine the influence of potential confounders.

**Methods:**

Patients who underwent hip arthroscopy or periacetabular osteotomy with preoperative International Hip Outcome Tool‐12 (iHOT‐12) scores and at least two follow‐up measurements at 6 months, 1 year, or 2 years were included from the UK Non‐Arthroplasty Hip Registry. iHOT‐12 score trajectories were analysed, and Latent Growth Curve Modelling was used to identify predictors of these trajectories.

**Results:**

Overall 9845 patients were included in this study. 7081 patients underwent a hip arthroscopy, and 1327 patients underwent a periacetabular osteotomy. For hip arthroscopy, there were significant improvements in the iHOT‐12 scores from baseline to 6 months, but no significant change from 6 months to 1 year. However, there was a decrease in the minimal clinically important difference from 1 to 2 year. For periacetabular osteotomy, there were significant improvements in the iHOT‐12 scores from baseline to 6 months, but no significant change from 6 months to 1 year, and from 1 to 2 years. Latent Growth Curve Modelling showed that body mass index (BMI) and sex had a significant impact on pre‐operative iHOT‐12 scores, while age and sex significantly influenced the recovery slope.

**Conclusions:**

Patients who underwent hip preservation surgery exhibited significant improvement in iHOT‐12 scores, surpassing the minimal clinically important difference at 6 months postoperatively. This improvement plateaued by 2 years, with a slight decline in scores between 1 and 2 years following hip arthroscopy, though the decrease remained within the clinically meaningful range. BMI, age and sex influenced score trajectories, highlighting the importance of setting patient expectations pre‐operatively.

**Trial Registration:**

The UK's Non‐Arthroplasty Hip Registry https://www.nahr.co.uk/.

**Level of Evidence:**

Level III.

**Abbreviations**
BMI: body mass index
*EQ‐5D*
: EuroQol‐5‐Dimensions
FAI: femoroacetabular impingement
HA: hip arthroscopy
iHOT‐12: International Hip Outcome Tool‐12
iHOT‐33: International Hip Outcome Tool‐33
LGCM: latent growth curve modelling
MCID: minimal clinically important difference
NAHR: Non‐Arthroplasty Hip Registry
PAO: periacetabular osteotomy
PROMs: patient‐reported outcome measures
RECORD: Reporting of Studies Conducted using Observational Routinely‐collected Health Data
STROBE: Strengthening the Reporting of Observational Studies in Epidemiology
THA: total hip arthroplasty

## INTRODUCTION

Hip arthroscopy (HA) for femoroacetabular impingement (FAI) and periacetabular osteotomy (PAO) for hip dysplasia are commonly performed to improve the quality of life, preserve the hip joint and delay arthroplasty in young adults [35]. Significant improvements in patient‐reported outcome measures (PROMs) after both HA and PAO have been reported, highlighting the effectiveness of hip preservation surgery [14, 15, 16].

The International Hip Outcome Tool‐12 (iHOT‐12) is a validated, joint specific outcome measure used for young adults with hip pathology [14, 15]. Developed in Canada, the USA, and the UK in 2012, it is a shortened version of the International Hip Outcome Tool‐33 (iHOT‐33). Its simplicity makes it suitable for both initial assessments and postoperative follow‐up in routine clinical practice [13, 14, 25, 28, 37, 39]. Due to its simplicity, it has been reported to be useful for initial assessment and postoperative follow‐up in routine clinical practice [13].

Accurately predicting the post‐operative trajectory of PROMs is crucial for patient selection and setting expectations at the pre‐operative consultation, as patients generally expect continuous improvement in pain relief, satisfaction, and function from the immediate post‐operative period until a stable plateau [6, 32, 36]. High‐quality data on PROM progression and the factors influencing their trajectory after hip preservation surgery enhance the evaluation of surgical indications, patient selection, and postoperative outcomes; however, prior studies have been limited by small, single‐surgeon cohorts, reducing generalisability [6, 8, 33].

This study aimed to evaluate the trajectory of PROMs up to two years after hip preservation surgery using the UK's Non‐Arthroplasty Hip Registry (NAHR) dataset and to identify factors influencing this trajectory. We hypothesised that PROMs would improve postoperatively and plateau over time, and that demographic or clinical variables may influence this pattern.

## METHODS

### Study design

This study was a Registry study based on the NAHR dataset. Approval for this observational study was granted by the NAHR steering committee (reference NAHR/2023/10). This study involved a retrospective analysis of data from a prospective database encompassing a consecutive series of hip preservation surgery and is reported according to The Reporting of Studies Conducted using Observational Routinely‐collected Health Data (RECORD) extension [4] of the Strengthening the Reporting of Observational Studies in Epidemiology (STROBE) guidelines for reporting of observational studies [40].

The establishment of the NAHR in 2012 aimed to gather comprehensive data at a national level regarding patients who were undergoing hip preservation surgery in the UK [18]. The registry encompasses data provided by 111 surgeons practising in 120 hospitals. Now in its 12th year, it has data of 22,872 pathways and is regarded as one of the largest registries in the arena of hip preservation [34]. The NAHR collects specific data including patient demographics, diagnoses, operative details, and PROMs: the EuroQol‐5‐Dimensions (EQ‐5D) [31] and iHOT‐12. In this study, hip preservation surgery was defined as either hip arthroscopy (HA) for femoroacetabular impingement (FAI) or periacetabular osteotomy (PAO) for developmental dysplasia of the hip (DDH). Although the NAHR also collects data on other joint‐preserving procedures such as open surgery for FAI and femoral osteotomy, these were excluded from the current analysis.

### Data sources/measurement

The pre‐operative data included in this study were collected directly from patients by their respective surgical teams and submitted to the NAHR. The post‐operative data at time points of 6 months, 1 year and 2 years is collected via an email link which is directly sent to the patient by the database automatically, leaving the clinician out of the process of post‐operative data collection. NAHR data submission is voluntary. The primary outcome is the iHOT‐12.

### Participants

Eligibility criteria for inclusion in this study were as follows: (1) Patients who were included the NAHR undergoing hip preservation surgery between 28 May 2012 and 1 March 2022, (2) patients who were 14 years or older at the time of inclusion in the registry, (3) patients who had a pre‐operative iHOT‐12 score and (4) who had iHOT‐12 scores recorded for at least two of the following three timepoints: 6 months, 1 year, and 2 years. Patients who underwent concurrent PAO and HA were included in the PAO cohort.

### Variables

From the registry, information regarding patient age, body mass index (BMI), sex, specific surgical procedure(s), operative side, presence of FAI, presence of arthroscopic acetabular cartilage damage and total iHOT‐12 scores at all accessible timepoints were extracted. Demographic variables were assessed to ensure data quality. Any data that was inaccurate, such as surgery dates falling outside the registry's timeframe or negative age/BMI values, was eliminated from the analysis on a complete‐case basis.

### Sources of bias

Given that not all patients had sufficient data for analysis, there is a potential for either systematic or random bias to have been introduced. For this reason, demographic characteristics for the cohort not included in the study due to missing data are also presented. The registry data does have areas of incomplete datasets. Excluding all incomplete data would result in responder bias, therefore, we included cases with a minimum of two post‐operative scores. Sensitivity analysis comparing patients with complete iHOT‐12 data across all four time points and those with data from at least two time points showed no statistically significant differences at any time point (all *p* > 0.05).

### Study size

The required sample size was calculated to detect a difference between time points using G*Power software (Universitat Kiel, Germany). To ensure that even small but significant changes in PROMs would not be overlooked assuming a small effect size (*η* ^2^ = 0.01). The sample size calculation considered an α value of 0.01 and a power of 95%. With these parameters, a sample size of 214 patients was determined to be necessary for adequate power in a repeated measures analysis of variance with two groups (HA or PAO) and four measurements.

### Quantitative variables

Demographic variables are reported descriptively, as mean (standard deviation) or frequencies and proportions, as appropriate. Specifically, age is reported in years at the time of operation, BMI is reported in kg/m^2^, and sex, operative side, the presence of FAI and acetabular cartilage damage (defined as the presence of cartilage lesions observed intraoperatively during hip arthroscopy) are reported as frequencies and proportions. iHOT‐12 scores are reported as mean (standard error) at each timepoint. Data accuracy was verified by the first author (JY) and a co‐author (SE). Any unclear or ambiguous entries were reviewed and resolved in consultation with senior co‐authors (AM and VK).

### Statistical methods

Statistical analyses were performed using SPSS Version 28 (IBM, Armonk, New York). *p* < 0.05 was considered statistically significant. All variables were screened for normality using the Shapiro–Wilk test. Descriptive statistics were performed for all continuous variables.

A plateau in trajectory refers to the initial timepoint after surgery where there is no improvement or decline in iHOT‐12 scores beyond the minimal clinically important difference (MCID) compared to the previous timepoint. MCID was defined as a statistically significant difference of at least 13 points between timepoints, based on the previously established threshold [25]. A linear mixed model with a post hoc Bonferroni method was conducted to determine differences in the iHOT‐12 scores at each time point. Effect sizes (Cohen's *d*) and 95% confidence intervals (CIs) were also calculated to quantify the magnitude and precision of observed differences.

To analyse the characteristics of the trajectory after hip preservation surgery, latent growth curve modelling (LGCM) was performed using JASP (JASP Team, 2023, JASP version 0.18.1) [38]. In an LGCM, change is modelled as a function of time and is represented through the specification of latent variables referred to as growth factors [11]. A latent intercept and a latent slope are estimated based on the individual trajectories. Growth factors provide an estimate of the average trajectory and individual variation around that trajectory [11]. This model specification allows the estimated intercepts and slopes of the models to represent the overall change from pre‐operative to 2‐year follow‐up. Using LGCM, it is possible to estimate whether factors influence the slope or intercept of the trajectory. We investigated whether factors such as age, BMI, sex, the presence of FAI, and acetabular cartilage damage affect the post‐operative trajectory. In addition to statistical significance, standardised regression coefficients (*β*) and their 95% CIs were reported for LGCM to assess the strength and precision of associations between covariates and outcome trajectories.

## RESULTS

### Participants

**Figure 1:**
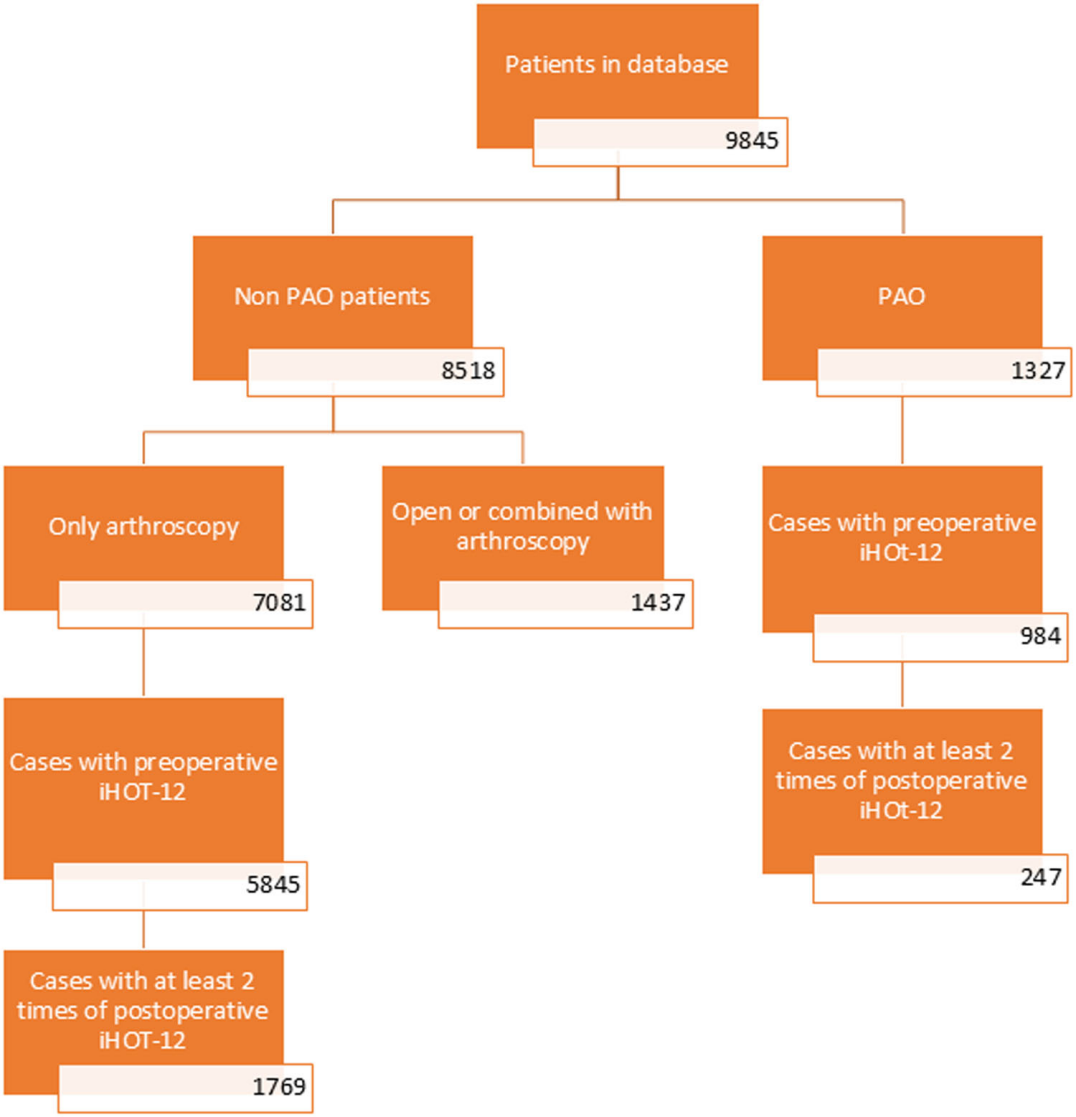
Flow chart demonstrating patient selection based on eligibility. iHOT‐12, the International Hip Outcome Tool‐12; PAO, periacetabular osteotomy.

Overall, we had 9845 eligible patients in this study. 8581 patients underwent hip preservation surgery without PAO, 7081 of whom underwent HA for FAI and/or labral tear, and 1437 patients underwent open or combined surgery with HA. 1327 patients underwent PAO. Figure 1 demonstrates the patient inclusion flow chart. HA patients with pre‐operative iHOT‐12 scores were 5845 and 1769 who had at least 2 post‐operative iHOT‐12 scores recorded, while PAO patients with pre‐operative iHOT‐12 scores were 984 and 247 patients who had the at least 2 post‐operative iHOT‐12 scores recorded. Table 1 summarises demographic characteristics of included and excluded patients in both groups.

**Table 1:** Baseline characteristics of patients included and excluded from analysis.

	HA included (*N* = 1769)	HA excluded (*N* = 5312)	PAO included (*N* = 247)	PAO excluded (*N* = 1080)
Age, mean (SD) (years)	36.3 (10.8)	34.9 (11.2)	36.1 (10.4)	35.6 (11.3)
BMI, mean (SD) (kg/m²)	25.8 (4.4)	26.0 (7.5)	25.7 (5.2)	25.7 (4.6)
Female (%)	1137 (64.3)	3051 (57.4)	164 (66.4)	679 (62.9)
Male (%)	632 (35.7)	2257 (42.5)	83 (33.6)	400 (37.0)
Right side surgery, *n* (%)	991 (56.0)	2928 (55.1)	143 (57.9)	585 (54.2)
Left side surgery, *n* (%)	763 (43.1)	2208 (41.6)	98 (39.7)	433 (40.1)
FAI present, *n* (%)	1319 (74.6)	3305 (62.2)	145 (58.7)	438 (40.6)
Acetabular cartilage damage, *n* (%)	587 (33.2)	1790 (33.7)	N/A	N/A

Abbreviations: BMI, body mass index; FAI, femoroacetabular impingement; HA, hip arthroscopy; PAO, periacetabular osteotomy; SD, standard deviation.

### The trajectory of PROMs after HA and PAO

**Figure 2:**
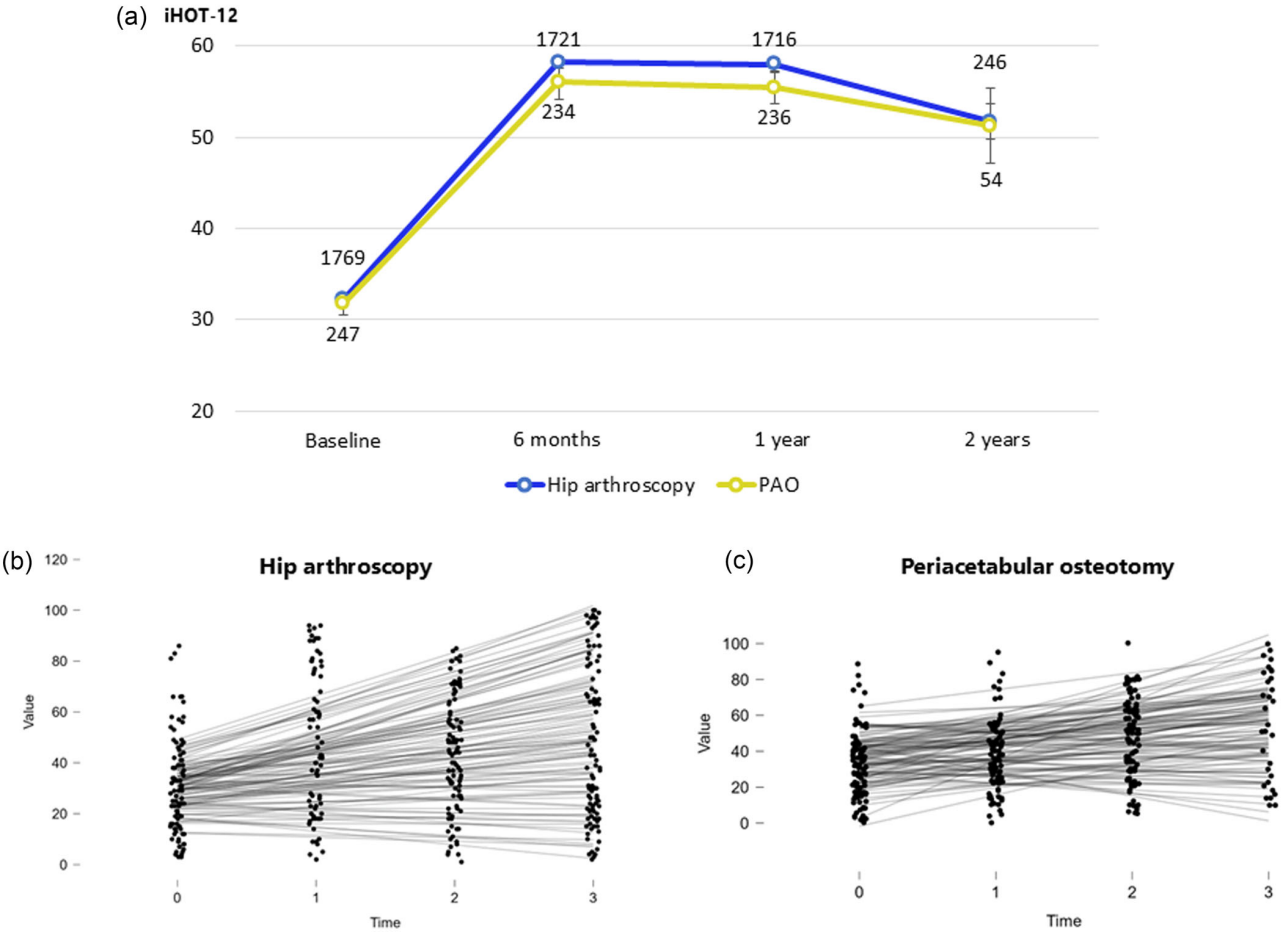
(a) Trajectory of iHOT‐12 after HA and PAO. Error bars represent the standard error of the mean. The number in the figure means the total number of patients with iHOT‐12 scores at each time point. (b) The linear trajectory of iHOT‐12 after HA by LGCM. (c) The linear trajectory of iHOT‐12 after PAO by LGCM. HA, Hip arthroscopy; iHOT‐12, International Hip Outcome Tool‐12; LGCM, latent growth curve modeling; PAO, periacetabular osteotomy.

The trajectory of iHOT‐12 after HA and PAO is represented in Figure 2. For patients undergoing HA, the mean pre‐operative iHOT‐12 Score was 32.2 (0.4). There were statistically significant interval improvements in iHOT‐12 scores of HA patients from baseline to 6 months (mean: 58.2 [0.7]), with a large effect size (Cohen's *d* = –0.93; 95% CI: –29.54 to –20.76). From 6 months to 1 year, there was no statistically significant change (mean: 58.0 [0.7]), with a small effect size (Cohen's *d* = 0.10; 95% CI: –1.56 to 6.57). From 1 year to 2 years, there was also no significant change (mean: 51.7 [1.9]), and the effect size was negligible (Cohen's *d* = 0.005; 95% CI: –4.41 to 4.69). The changes from baseline to 6 months, 1 year and 2 years were more than the threshold for a clinically meaningful change based on an MCID value of 13.0, and the change from 1 year to 2 years of iHOT‐12 scores was below the MCID value of 13.0. Among patients undergoing PAO, the mean pre‐operative iHOT‐12 Score was 31.7 (1.2). There were statistically significant interval improvements in iHOT‐12 scores from baseline to 6 months (mean: 56.0 [1.8]), with a large effect size (Cohen's *d* = 1.01, 95% CI: 16.29 to 35.31). However, there were no significant changes from 6 months to 1 year (mean: 55.4 [1.7]; Cohen's *d* = –0.11, 95% CI: –10.63 to 5.77) or from 1 year to 2 years (mean: 51.3 [4.2]; Cohen's *d* = 0.07, 95% CI: –8.77 to 12.51). The changes from baseline to 6 months, 1 year and 2 years were more than the threshold for a clinically meaningful change based on an MCID value of 13.0.

Figure 2b,c show the individual differences in the linear trajectory of HA and PAO by LGCM. As for HA trajectory, the mean estimate of intercept was 46.7 and the variance 59.2 (*p* < 0.001 and *p* = 0.016, respectively) and the mean estimate of the linear slope was 17.9 and the variance 77.8 (*p* < 0.001), which implies that the average intersect of the trajectory was 46.7, and there was a positive average linear growth rate over time. The estimated mean linear slope was 17.9, with a p‐value lower than 0.001. Therefore, there is a significant average linear increase of 17.9 in iHOT‐12 scores across 2 years (Table 2). The latent curve of PAO trajectory showed the mean estimate of intercept was 55.8 and the variance −77.8 (*p* < 0.001 and *p* = 0.234, respectively) and the mean estimate of the linear slope was 13.0 and the variance −5.0 (*p* = 0.008 and *p* = 0.850, respectively, Table 2). As negative variances are theoretically inadmissible, these were considered boundary estimates resulting from estimation limitations in the LGCM process and interpreted with caution.

**Table 2:** The results of the latent curve in HA and PAO.

					95% Confidence interval	
	Component	Parameter	Estimate	*p* value	Lower	Upper
HA	Intercept	Mean	46.7	<0.001	41.01	52.49
		Variance	59.2	0.016	11.14	107.33
	Linear slope	Mean	17.9	<0.001	13.62	22.18
		Variance	77.8	<0.001	49.8	105.71
PAO	Intercept	Mean	55.8	<0.001	41.0	70.52
		Variance	−77.8	0.234	−206.1	50.43
	Linear slope	Mean	13.0	0.008	3.38	22.54
		Variance	−5.0	0.85	−56.19	46.29

Abbreviations: HA, hip arthroscopy; PAO, periacetabular osteotomy.

### Predictive coefficient to the trajectory

In the regression coefficients table, BMI and sex showed significant correlations with the intercept in the HA trajectory (*p* < 0.001), while age and sex exhibited significant correlations with the linear slope (*p* = 0.002, *p* < 0.001, respectively) (Table 3). The estimate of BMI on the intercept was −0.64, which means higher BMI patients have lower iHOT‐12 scores at baseline, while the estimate of sex on the intercept was 8.23, which means male patients are likely to have higher iHOT‐12 scores at baseline (Table 3). The estimate of age on the linear slope was −0.073, suggesting that the improvement in post‐operative iHOT‐12 scores is more gradual in older individuals than younger patients. The estimate of sex on the linear slope was −3.17, which implies that female patients exhibit a steeper linear growth trend than male patients. Since the p‐value of the regression coefficient from acetabular cartilage damage and the presence of FAI to the intercept and the linear slope were more than 0.05, both factors were not significant predictors in explaining the linear growth trend of the trajectory.

**Table 3:** The regression coefficient of predicting factors for the trajectory after HA and PAO.

					95% Confidence interval	
	Component	Predictor	Estimate	*p* value	Lower	Upper
HA	Intercept	Age	0.07	0.119	−0.02	0.15
		BMI	−0.64	<0.001	−0.84	−0.44
		Male	8.23	<0.001	6.35	10.11
		Acetabular cartilage damage	−0.90	0.379	−2.74	1.04
		FAI	1.55	0.152	−0.57	3.68
	Linear slope	Age	−0.07	0.02	−0.14	−0.01
		BMI	−0.07	0.335	−0.23	0.08
		Male	−3.17	<0.001	−4.57	−1.76
		Acetabular cartilage damage	−0.21	0.77	−1.63	1.21
		FAI	1.31	0.108	−0.29	2.9
PAO	Intercept	BMI	−0.64	0.013	−1.14	−0.13
		Age	−0.10	0.478	−0.39	0.18
		Male	6.26	0.027	0.71	11.80
	Linear slope	BMI	−0.17	0.314	−0.5	0.16
		Age	0.09	0.413	−0.12	0.29
		Male	−4.49	0.02	−8.27	−0.71

Abbreviations: BMI, body mass index; FAI, femoroacetabular impingement; HA, hip arthroscopy; PAO, periacetabular osteotomy.

As for PAO trajectory, BMI and sex exhibited significant correlations with the intercept (*p* = 0.013 and 0.027, respectively), while sex showed a significant correlation with the linear slope (*p* = 0.020) (Table 3). The estimates of BMI and sex on the intercept were −0.64 and 6.26, respectively. Additionally, the estimate of sex on the linear slope was −4.49, indicating that the influence of BMI and sex is similar to that observed in the HA trajectory (Table 3).

## DISCUSSION

The most important finding of this study is that patients who underwent hip preservation surgery showed an improvement in PROMs exceeding the MCID at 6 months postoperatively, which then plateaued until 2 years postoperatively, despite a decrease in iHOT‐12 scores from 6 months to 2 years. Factors influencing the trajectory of both HA and PAO included BMI, age, and sex. LGCM revealed that BMI and sex significantly affected the pre‐operative PROMs of patients undergoing HA and PAO, whilst age and sex significantly influenced the recovery slope after HA or PAO. This knowledge, obtained from a large registry dataset with over 110 surgeons contributing, enables surgeons to improve patient selection and also to explain to patients the expected course of improvement following hip preservation surgery in the shorter term. *Importantly, a plateau in PROMs does not necessarily indicate full recovery, but rather stabilisation in measurable improvement.* It is important to understand when improvement plateaus after hip preservation surgery, as this knowledge directly impacts decisions on the appropriate duration of postoperative rehabilitation and follow‐up. While hip preservation surgery has demonstrated favourable outcomes in many reports [14, 15, 16], there are also patients who do not experience significant improvement [22, 23]. Therefore, if patients with risk factors for poor postoperative progress can be identified preoperatively, alternative treatment strategies—such as continued non‐operative rehabilitation or even primary total hip arthroplasty (THA)—should be considered. Our findings may aid clinicians in stratifying patients and optimising treatment selection based on predicted recovery trajectories.

To better contextualise our results within existing literature, we considered previous studies examining longer‐term outcome patterns. Previous work found that most patients after arthroscopic treatment of FAI achieved MCID by 6 months postoperatively, with the proportion increasing up to 2 years [29]. Röling et al. identified two subgroups with distinct functional recovery trajectories after HA: improvers and non‐improvers [33]. In a recent study using latent class modelling, Bodendorfer et al. identified that approximately 22% of patients followed a 'late regressor' trajectory, characterised by initial improvement but subsequent decline in PROMs between one and two years postoperatively. Risk factors for this group included psychiatric history, chronic pain, and workers’ compensation status [5]. This suggests that the recovery trajectory is not always linear, and a subset of patients may demonstrate delayed decline despite initial improvement.

In our registry study, we also observed a modest decline in iHOT‐12 scores between one and two years postoperatively, which may partly reflect the presence of such 'late regressor' patients with suboptimal recovery trajectories. This highlights the need for long‐term follow‐up and further attention to modifiable preoperative factors.

As a potential cause of the decline in post‐operative PROMs, incorrect patient selection for surgery may be considered as the primary factor. Several previous studies reported that patient factors such as older age, osteoarthritis, sex and elevated BMI have been associated with worse post‐operative outcomes [2, 7, 9, 12, 17, 20, 23, 24, 26, 27]. There is also a worry that conducting a joint‐preservation procedure in this specific subgroup might result in a rapid deterioration of their clinical condition, necessitating THA at an earlier stage [1, 41]. A study conducted using the Statewide Planning and Research Cooperative System database involving 3957 patients who underwent hip arthroscopy, revealed that obese patients had a more than five‐fold higher likelihood of needing to undergo conversion to THA [20]. In our study, LGCM revealed that BMI, sex and age significantly affected the trajectory after HA or PAO. Therefore, incorrect or overly broad surgical indications for hip preservation treatment may have caused the progression of osteoarthritis in some patients, leading to a decline in post‐operative PROMs.

Beck et al. reported that pre‐operative isometric hip strength measurements, particularly abduction and extension, exhibit a positive correlation with the 6‐month post‐operative hip function score after HA [3]. Jacobs et al. evaluated sex‐based differences in hip abductor function in relation to lower extremity landing kinematics and demonstrated that females tend to have lower hip abductor isometric peak torque [19]. These findings suggest the potential involvement of muscle strength in the changes observed in post‐operative PROMs following HA. A recent systematic review and meta‐analysis demonstrated that prehabilitation programs are associated with improvements in pre‐operative outcomes for patients undergoing various orthopaedic surgical procedures [21, 30]. These findings suggest that muscle strength and conditioning before and after surgery may also influence longer‐term outcomes, and may help mitigate the decline observed in some patients between one and two years postoperatively.

## LIMITATIONS

NAHR does not mandate data submission, introducing potential bias since contributions are voluntary for surgeons and institutions. Second, the small patient number at the 2‐year mark could lead to attrition bias. The LGCM analysis assumed linearity, risking oversight of non‐linear post‐operative trajectories. Third, the observed decline in iHOT‐12 scores from 1 to 2 years in the HA group may not be fully captured. Therefore, we used line graphs to visually examine mean value trends. Another limitation is the presence of missing data in longitudinal follow‐up. To address this, we included patients with at least two iHOT‐12 scores across the four time points. This approach is consistent with previous study [10]. Additionally, we performed a sensitivity analysis comparing patients with complete data at all four time points and those meeting the two‐time‐point inclusion criterion. The analysis showed no significant differences in the trajectory of iHOT‐12 scores between the two groups, suggesting that the inclusion of patients with partially missing data did not bias the results. However, we acknowledge that this may limit the interpretation of individual recovery trajectories, and it introduces a risk of bias from incomplete data, which has been addressed as a limitation. Finally, although more frequent follow‐up intervals or longer‐term follow‐up could provide further insight, these approaches are limited by the nature of registry data, where the completeness of data declines with both increasing follow‐up duration and shorter intervals between measurements.

## STRENGTHS

First and foremost, it relies on data obtained from a nationwide registry, which encompasses information from a diverse group of surgeons and hospitals with varying levels of expertise and experience, as well as varying numbers of cases handled. Moreover, our study involves a substantially large number of patients who underwent hip preservation surgery, including both HA and PAO.

## CONCLUSIONS

Patients who underwent hip preservation surgery exhibited an improvement in iHOT‐12 scores that exceeded the MCID at six months post‐operatively. There is a plateau in the trajectory of PROMs of hip preservation surgery after 6 months until 2 years. Factors influencing the trajectory of both HA and PAO included BMI, age, and sex. LGCM revealed that BMI and sex significantly affected the pre‐operative PROMs of patients undergoing HA and PAO, while age and sex significantly influenced the recovery slope after HA or PAO. These findings are highly valuable in improving patient selection, effective communication with patients and setting expectations prior to surgery.

## AUTHOR CONTRIBUTIONS

**Junya Yoshitani**: Conceptualisation; data curation; investigation; writing–original draft; writing–review and editing. **Seper Ekhtiari**: Writing–review and editing. **Ajay Malviya**: Data curation; writing–review and editing. **Vikas Khanduja**: Conceptualisation; supervision; writing–review and editing; overall responsibility.

## CONFLICT OF INTEREST STATEMENT

Mr. Khanduja is a Consultant to Smith and Nephew, Stryker and Arthrex. He is the Associate Editor in Chief to JISAKOS. On the Board for SICOT, BOA & ISHA. Other authors declare no conflict of interest.

## ETHICS STATEMENT

The UK's Non‐Arthroplasty Hip Registry (NAHR) steering committee approved this retrospective cohort study of prospectively collected data. Approval for this observational study was granted by the NAHR steering committee (reference NAHR/2023/10).

## Data Availability

The data set in this registry study will be available via NAHR steering committee.
